# Napkin-ring sign plaques are associated with clinical outcome in patients with acute ischemic stroke after endovascular therapy

**DOI:** 10.3389/fneur.2026.1792239

**Published:** 2026-06-11

**Authors:** Linkao Chen, Rui Huang, Taotao Tao, Chengfei Zhu, Xiaohua Li, Xinwei He

**Affiliations:** 1 Graduate School, ZheJiang Chinese Medical University, Hangzhou, Zhejiang, China; 2 Department of Neurology, Taizhou Central Hospital (Taizhou University Hospital), Taizhou University, Taizhou, Zhejiang, China

**Keywords:** acute ischemic stroke, clinic outcome, endovascular therapy, large-vessel occlusion, napkin-ring sign plaque

## Abstract

**Objectives:**

Few studies have investigated the association between the carotid artery napkin ring sign (NRS) and acute ischemic stroke (AIS). This study investigated whether the burden of carotid artery NRS plaques affects the outcomes of patients with AIS requiring endovascular therapy (EVT).

**Methods:**

This retrospective, single-center study enrolled patients with large-vessel occlusion of the anterior circulation. Plaques were evaluated using preoperative cervicocerebral computed tomography angiography. Successful arterial recanalization was defined as a modified thrombolysis score of 2b to 3 for cerebral infarction on the final angiographic examination. A poor outcome was defined as a modified Rankin scale (mRS) score >2 at 3 months after stroke. We used univariate and logistic regression analyses to assess NRS plaque characteristics and the association between NRS plaques and revascularization and functional outcomes in patients with AIS requiring endovascular therapy.

**Results:**

Higher total NRS plaques and areas were observed on the symptomatic side than on the contralateral side. Patients with poor outcomes had a high percentage of total NRS plaques on both symptomatic and contralateral sides in the univariate analyses. NRS plaque areas were significantly high in patients with poor outcomes and showed good diagnostic accuracy in discriminating the presence of a poor outcome on both sides. After adjusting for other covariates, the association between the NRS area and poor outcome remained statistically significant.

**Conclusion:**

We documented that the presence and areas of NRS plaques on cervicocerebral CTA are associated with reduced recanalization, increased symptomatic intracranial hemorrhage (sICH), and poor outcomes in patients with AIS after EVT.

## Introduction

Acute ischemic stroke (AIS) is the most common form of stroke, accounting for 80% of cases (1). Large-vessel occlusion (LVO) associated with atherosclerotic disease accounts for 10–65% of LVO strokes and is more frequent in people of Asian, Hispanic, or Black ethnicities (2, 3). Recanalization of LVO using endovascular therapy (EVT) is an effective treatment; however, there is still a lack of effective management strategies and prediction methods.

In general, vulnerable plaques are characterized by features, such as intraplaque hemorrhage, a thin or ruptured fibrous cap overlying a large lipid-rich necrotic core, inflammation, neovascularization, and an ulcerated or fissured surface (4, 5). These features, called high-risk plaques, are associated with a high risk of ischemic stroke and are independent of the degree of carotid artery narrowing. A high-density ring structure around a low-density center, described as the napkin ring sign (NRS) (6), is considered a sign of cardiovascular and cerebrovascular diseases (7).

Multiple noninvasive imaging methods, such as ultrasound, computed tomography (CT), and magnetic resonance imaging (MRI), can detect NRS plaques (8). MRI is the most suitable technique for characterizing NRS (9, 10); however, it is difficult for patients with AIS to cooperate. By contrast, CT angiography (CTA) is a good noninvasive modality for the detection of vulnerable plaques with similarly high diagnostic accuracy, and it is easier for patients to tolerate (11). On the other hand, ultrasound has shown low accuracy in evaluating carotid plaques. Although studies suggest that multimodal ultrasound scoring systems can help identify vulnerable plaques, these systems are still not widely used (12, 13). Therefore, we prefer CTA for characterizing carotid plaque morphology in patients with AIS.

We initially observed in a study of coronary CTA that fibrous tissue necrosis surrounding the lipid core of the NRS may increase the incidence of major adverse cardiac events (14). A previous study reported that an increased incidence of NRS plaques was an independent and important risk factor for AIS (15). Currently, data regarding the effect of NRS plaques on clinical outcomes after EVT are limited. Therefore, the present study hypothesized that a high incidence of NRS plaques would lead to worse recanalization and high mortality in patients undergoing EVT and would be positively correlated with NRS plaque area.

## Materials and methods

### Patient recruitment

The studies involving human participants were reviewed and approved by the Ethics Committee of our Hospital [2024 L-11-81]. Written informed consent for participation was not required for this study, in accordance with national legislation and institutional requirements.

We retrospectively reviewed the EVT database maintained at our Hospital from January 2018 to May 2024. The anesthesia methods, materials, and techniques were selected by specialized neurointerventional physicians.

Patients with anterior circulation large-vessel occlusion (ICA, middle cerebral artery segments M1 and M2) on preoperative head and neck CTA were included.

The exclusion criteria were as follows: (1) pre-admission modified Rankin scale (mRS) score > 2, (2) history of cerebrovascular disease with obvious sequelae, (3) common carotid artery occlusion, (4) prior carotid endarterectomy or stenting, (5) severe systemic illnesses, (6) incomplete clinical baseline data, and (5) poor quality or missing images.

### EVT and clinical outcome

After successful local anesthesia, the femoral artery was punctured to determine the location of arterial occlusion. The microcatheter was guided to the distal end of the thrombus, the thrombus stent was placed at the thrombus position, and the stent was slowly removed to remove the thrombus. Alternatively, intermediate catheter aspiration combined with stent thrombus removal was performed. Multiple procedures were performed if necessary, and recanalization was determined using angiography. Revascularization was assessed at the end of surgery using the modified cerebral infarction thrombus (mTICI) score. An mTICI score of 2b to 3 is considered a sign of success (16).

Symptomatic intracranial hemorrhage (sICH) was defined according to the European Cooperative Acute Stroke Study III criteria (17). The clinical outcomes were assessed at 3 months using an mRS score ranging from zero (complete independence) to six (death). The assessments were conducted by a certified assessor. Poor functional outcomes were defined as mRS scores of > 2 (18).

### Vascular risk factors and TOAST classification system

Demographic and clinical characteristics, as well as vascular risk factors, were extracted from the patients’ medical records. The risk factors were defined as follows: hypertension (repeated systolic/diastolic blood pressure > 140/90 mmHg or a history of hypertension), diabetes (based on symptoms, such as polydipsia, polyuria, unexplained weight loss, a random intravenous blood glucose concentration ≥ 11.1 mmol/L, fasting blood glucose concentration ≥ 7.0 mmol/L, 2-h glucose concentration ≥ 11.1 mmol/L, glycosylated hemoglobin > 6.3%, self-reported diabetes, or use of oral hypoglycemic agents or insulin), hyperlipidemia (serum triglycerides > 1.7 mmol/L, low-density lipoprotein > 3.4 mmol/L, high-density lipoprotein cholesterol < 0.8 mmol/L, or use of statins), current smoking (actively smoking or having smoked for at least 6 months cumulatively), and alcohol consumption (more than 60 g of pure alcohol per day for men and 40 g per day for women) (19, 20).

The TOAST classification system includes five categories: (1) large-artery atherosclerosis (LAA), (2) cardioembolism, (3) small-artery occlusion, (4) stroke of other determined etiologies, and (5) stroke of undetermined etiology (21). Diagnoses were based on clinical features and data collected by tests, such as brain imaging (CT/MRI), cardiac imaging (including echocardiography), duplex imaging of extracranial arteries, arteriography, and laboratory assessments for a prothrombotic state.

### CTA protocols

A 64-slice Discovery CT750HD (GE, USA) was used with the following parameters: 100 kVp, 300 mAs, slice thickness of 0.625 mm; interval of 0.625 mm; and display field of view 250 mm × 250 mm. An intravenous iodized contrast agent (Iodol injection, 1.5–2 mL/kg; China Hengrui Pharmaceutical Co., Ltd.) was administered at an injection rate of 4.0 mL/s.

### CTA image analysis

**Figure 1:**
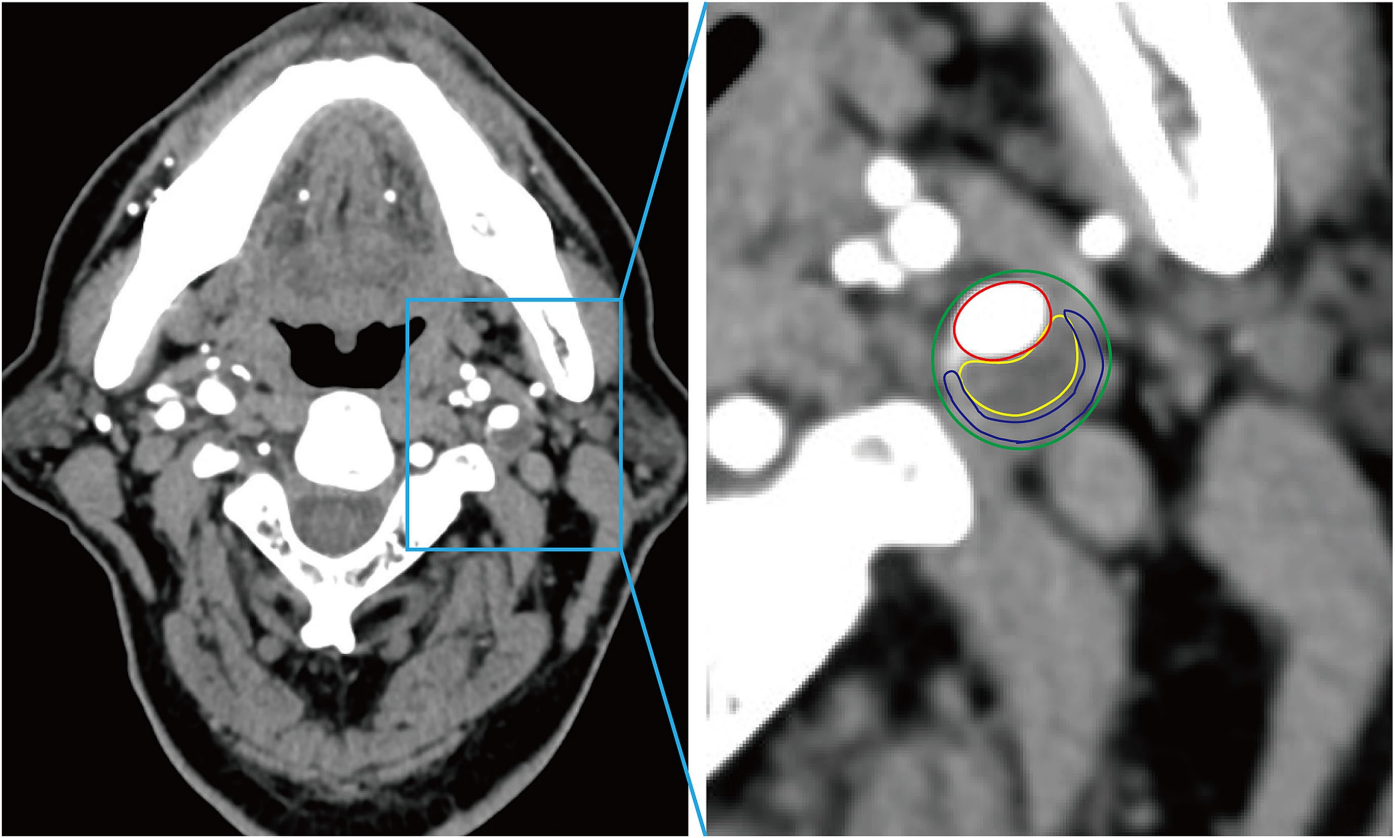
A 69-year-old man diagnosed with ischemic stroke in the left basal ganglia region with numbness and weakness in the right extremities had a plaque with NRS at the left common carotid artery identified on CTA. The yellow round demonstrates the low attenuation area of the plaque surrounded by a high-attenuation rim (blue round), and the red area represents the lumen field with contrast.

After scanning, all CT data were transferred to a post-processing workstation for image analysis. Reconstruction was performed on the axial, coronal, and sagittal planes, including volume rendering of different segments of the measured artery, maximum intensity projection, and multiplane and curved plane reformatting. An example is shown in Figure 1. To help readers better recognize the varying appearances of NRS, we have included imaging from two additional patients in the Supplementary material. These cases (Supplementary Figure S1) demonstrate plaques with a broader spectrum of density and size, illustrating how NRS can present in clinical practice.

Atherosclerotic lesions are distributed across certain segments (22). Because blood vessels in the intracranial segment are often occluded, we selected images of the bilateral common carotid arteries up to the initiation of the internal carotid artery for this study.

The NRS on CTA has been defined in previous studies as follows: an inner low-density core surrounded by an outer high-density ring of no more than 130 Hounsfield units [HU] (6, 23). All readings were performed with a fixed window setting (350 HU width, 50 HU level) (24). Additionally, when counting the area, select the largest section, and not near calcifications to avoid beam-hardening artifacts. The NRS area represents the total area on the ipsilateral side, calculated as the maximum cross-section if more than one NRS patch was present. Two independent neurologists, blinded to the patients’ clinical data, evaluated the areas. The intraclass correlation coefficient values for right- and left-sided scores from the two neurologists were 0.874 (*p* < 0.001) and 0.813 (*p* < 0.001), respectively. The average values from the two observers were used in the final analysis.

The remodeling index was calculated as the maximum narrow cross-sectional area of the NRS site with the greatest plaque burden divided by the average cross-sectional area of the proximal reference segment and the remote reference segment (≤ 10 mm). A remodeling index ≥ 1.05 indicates positive remodeling (PR) (25).

### Statistical analysis

Categorical data were presented as frequencies and percentages, and the chi-square test or Fisher’s exact test was used for comparison. Continuous data with a normal distribution are presented as mean ± standard deviation. Continuous data without a normal distribution are presented as median (interquartile range) and were compared using Student’s *t*-test or the Mann–Whitney U test. We calculated the interclass correlation coefficient and corresponding 95% confidence interval (CI) to evaluate inter-rater reliability. Receiver operating characteristic curve analysis was performed to assess the accuracy of NRS area in predicting poor outcomes. Multivariate analyses were performed using logistic regression models adjusted for potential influencing factors, which were selected based on univariate analyses (*p* < 0.05). All data were analyzed using SPSS 22.0 (IBM, Chicago, IL, USA).

## Results

### Patient characteristics

**Figure 2:**
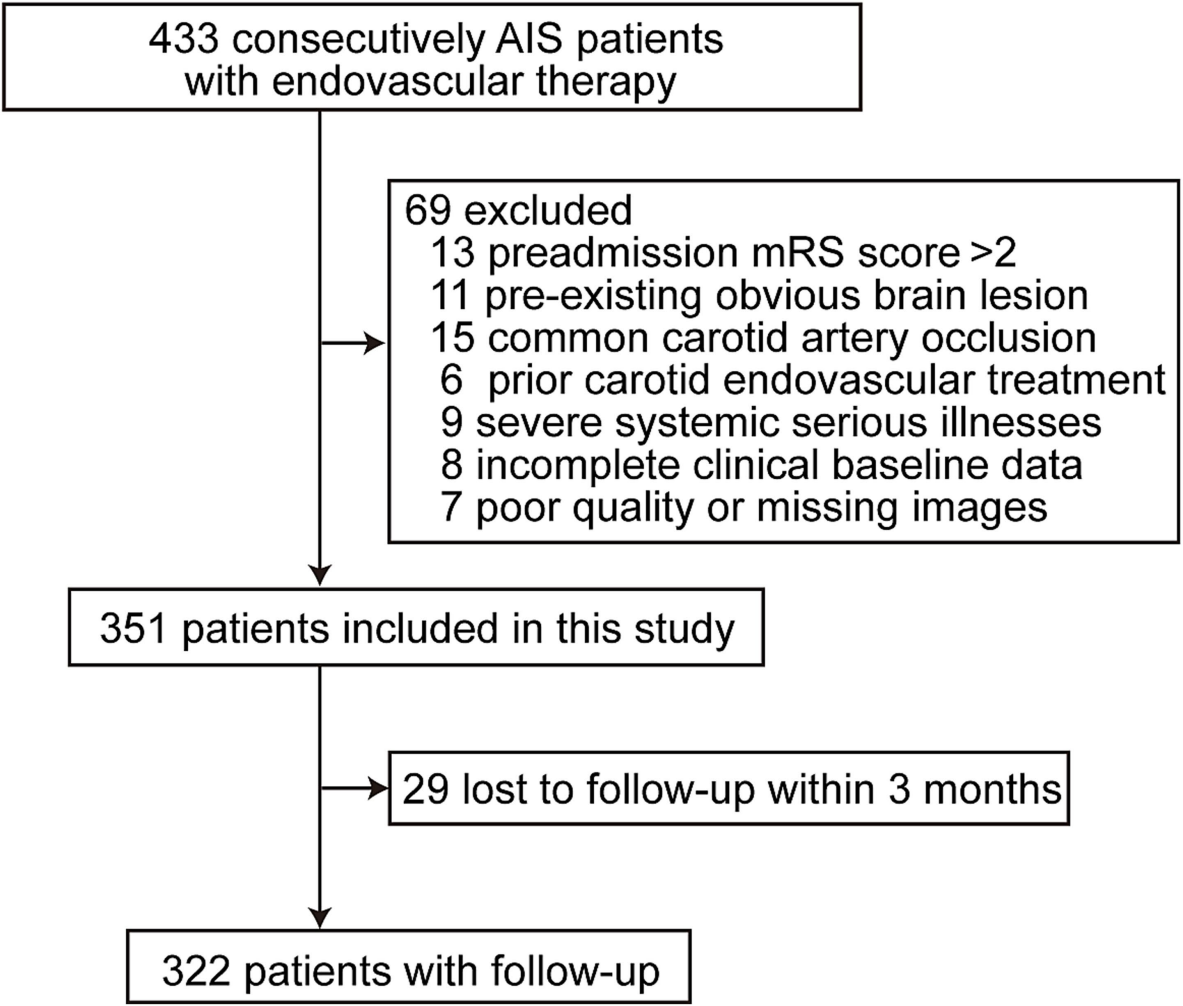
Study flow diagram. AIS, acute ischemic stroke.

Initially, 433 patients with AIS receiving EVT were screened, and after applying the aforementioned criteria, 351 patients were included in the analysis (Figure 2). The basic patient characteristics are summarized in Table 1. A total of 217 (61.8%) patients were men, the mean age was 69.4 ± 12.5 years, and the NIHSS score at baseline was 14 (9, 17). 97 (27.6%) patients had ICA occlusion, 231 (65.8%) had middle cerebral artery occlusion, and 23 (6.6%) had tandem lesions. According to the TOAST criteria, 152 (43.3%) patients were categorized as having LAA; 157 (44.7%) were attributed to cardioembolic sources, and 42 (12.0%) were assigned to other etiologies.

**Table 1:** Baseline characteristics of patients.

Variables	Total *N* = 351	Good outcome *N* = 129	Poor outcome *N* = 193	*P*
Age, years	69.4 ± 12.5	68.5 ± 12.5	70.7 ± 12.4	0.119
Male, n (%)	217 (61.8%)	72(55.8%)	121 (62.7%)	0.495
BMI	23.1 ± 5.5	22.8 ± 3.0	23.2 ± 3.3	0.275
Risk factors, *n* (%)				
Hypertension	224 (63.8%)	82 (63.6%)	126 (65.3%)	0.752
Diabetes mellitus	68 (19.4%)	33 (25.6%)	28 (14.5%)	0.301
Dyslipidemia	90 (25.6%)	34 (26.4%)	48 (24.9%)	0.764
Coronary heart disease	82 (23.4%)	24 (18.6%)	44 (22.8%)	0.366
Atrial fibrillation	157 (44.7%)	59 (45.7%)	85 (44.0%)	0.764
Stroke history	97 (27.6%)	38 (29.5%)	51 (26.4%)	0.551
Hypertension med use	132 (37.6%)	57 (44.2%)	74 (38.3%)	0.396
Diabetes med use	88 (25.1%)	34 (26.4%)	43 (22.3%)	0.401
Smoking	53 (15.1%)	22 (17.1%)	28 (14.5%)	0.536
Drinking	40 (11.4%)	15 (11.6%)	19 (9.8%)	0.610
Sit of occlusion				0.006
Internal carotid artery	97 (27.6%)	22 (17.1%)	64 (33.2%)	
Middle cerebral artery	231 (65.8%)	96 (74.4%)	117 (60.6%)	
Tandem lesion	23 (6.6%)	11 (8.5%)	12 (6.2%)	
Stroke evaluation				
NIHSS at baseline	14 (9, 17)	12 (9, 16)	15 (12, 18)	<0.001
Successful recanalization	322 (91.7%)	115 (89.1%)	178 (92.2%)	0.344
Onset to door time, min	181(103,306)	156(100,277)	187(102,299)	0.193
Onset to recanalization, min	330 (210, 480)	330 (195, 464)	360 (210, 480)	0.122
Intravenous thrombolysis	124 (35.3%)	56 (43.4%)	59 (30.6%)	0.018
Number of passes	2 (1, 3)	2 (1, 3)	2 (1, 3)	0.574
sICH, *n* (%)	36 (10.3%)	6 (4.7%)	29 (15.0%)	0.003
TOAST subtype, *n* (%)				0.392
Large-artery atherosclerosis	152 (43.3%)	51 (39.5%)	91 (47.2%)	
Cardioembolism	157 (44.7%)	63 (48.8%)	81 (42.0%)	
Others	42 (12.0%)	15 (11.6%)	21 (10.9%)	

Continuous data with a normal distribution are presented as mean ± standard deviation. Continuous data without a normal distribution are presented as median (interquartile range) and were compared using Student’s t-test or the Mann–Whitney U test. BMI, Body Mass Index; NIHSS, National Institutes of Health Stroke Scale, sICH, symptomatic intracranial hemorrhage, TOAST, Trial of Org 10,172 in Acute Stroke Treatment.

Among the patients with LAA, 93 (59.2%) had NRS plaques (51 patients had symptomatic NRS, 19 had contralateral NRS, and 23 had bilateral NRS). In the CE group, 76 (48.4%) patients had NRS plaques (37 patients had symptomatic NRS, 27 had contralateral NRS, and 12 had bilateral NRS). Patients with LAA had a higher percentage of NRS plaques than that of patients with CE (*p =* 0.030).

**Figure 3:**
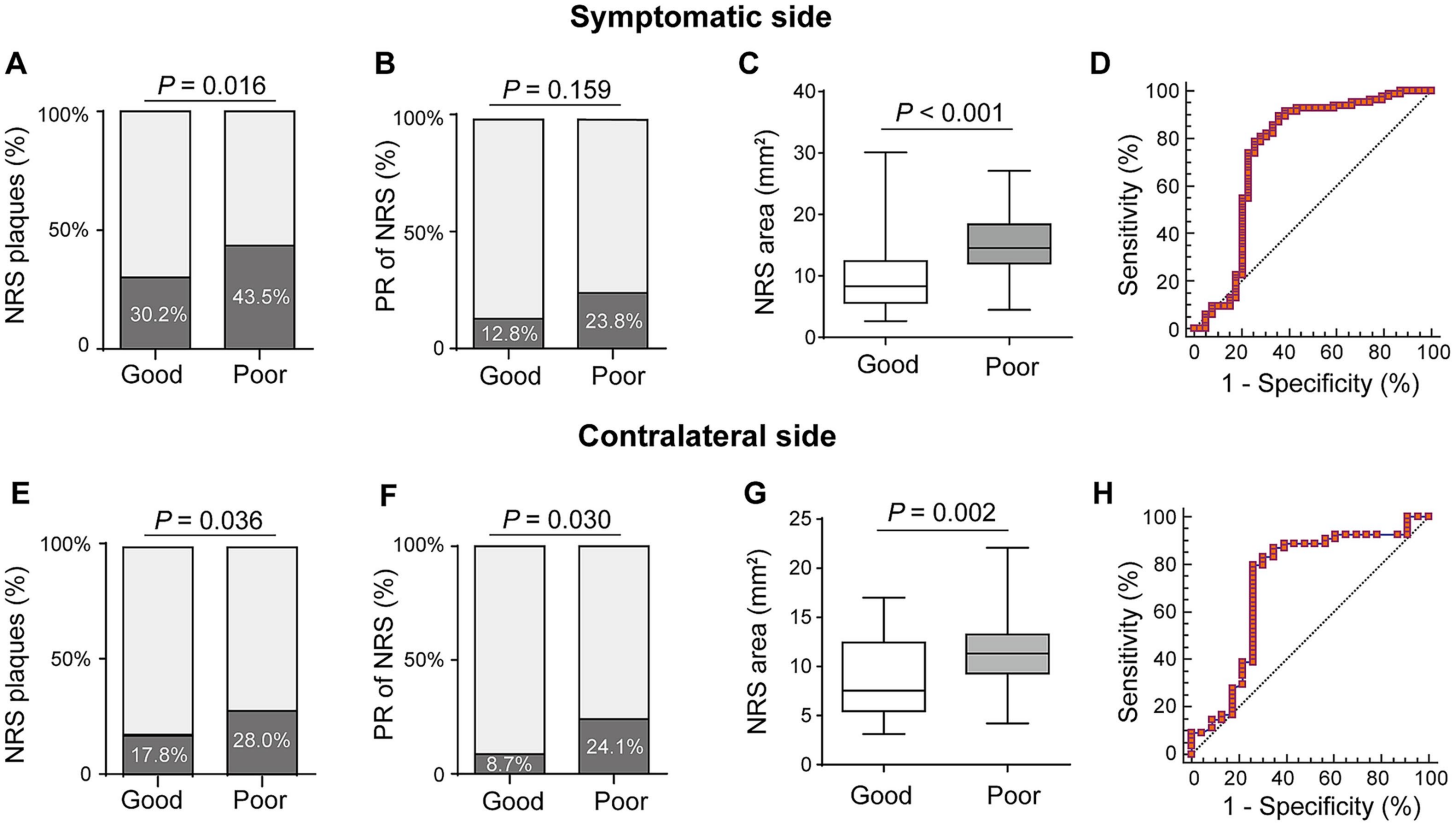
Association of napkin-ring sign plaques with 3-month functional outcome. **(A–D)** Comparison of patients with good or poor outcomes in symptomatic side. **(E–H)** Comparison of patients with good or poor outcomes in contralateral side. NRS, napkin-ring sign; PR, Positive remodeling.

The patients were followed up for 3 months, and 193 (59.9%) had poor outcomes. Patients with poor outcomes had a higher percentage of NRS plaques on the symptomatic side (the carotid artery ipsilateral to the ischemic stroke) (84 [43.5%] vs. 39 [30.2%], *p* = 0.016; Figure 3A) and the contralateral side (the opposite carotid artery) (54 [28.0%] vs. 23 [17.8%], *p* = 0.036; Figure 3E). In addition, a high percentage of PR was observed on the contralateral side (13 [24.1%] vs. 2 [8.7%], *p* = 0.030; Figure 3F)while there was no difference observed on the symptomatic side(20 [23.8%] vs. 5 [12.8%], *p* = 0.159; Figure 3B).

For NRS plaque area, a greater NRS area was observed on the symptomatic side (14.6 [12.1, 18.3] mm^2^ vs. 8.3 [5.7, 12.0] mm^2^*, p* < 0.001; Figure 3C) and on the contralateral side (11.3 [9.3, 13.2] vs. 7.5 [5.5, 10.8], *p* = 0.002; Figure 3G) (Table 2). Receiver operating characteristic curve analysis was conducted to assess the prognostic accuracy of the NRS area for poor outcomes. The area under the curve values were 0.747 (95% CI [0.660–0.821], *p* < 0.001; Figure 3D) with a cutoff of 9.13 mm^2^ on the symptomatic side and 0.723 (95% CI [0.609–0.819], *p* = 0.003; Figure 3H) with a cutoff of 9.71 mm^2^ on the contralateral side. After adjusting for age, sex, and differences between groups (including site of occlusion, NIHSS score at baseline, intravenous thrombolysis), NRS area was independently associated with poor functional outcome on both sides. On the symptomatic side, each unit increase in NRS area was associated with a higher likelihood of poor outcome (OR 1.285, 95% CI 1.150–1.435, *p* < 0.001). Similarly, on the contralateral side, NRS area remained a significant predictor (OR 1.260, 95% CI 1.033–1.536, *p* = 0.022) (Table 3). In contrast, the presence of NRS plaques and PR of NRS did not show statistically significant associations with poor outcome after adjustment for confounding factors on either side.

**Table 2:** Napkin-ring sign plaques in symptomatic side stratified by recanalization symptomatic intracranial hemorrhage and 3-month functional outcome.

Characteristics		NRS plaques		PR of NRS		NRS area	
		N (%)	*P*	N (%)	*P*	mm^2^	*P*
Symptomatic side							
Recanalization	Yes	114 (35.4%)	**0.005**	19 (16.7%)	**0.036**	12.6 (8.7, 16.7)	0.158
sICH	Yes	23 (63.9%)	**0.001**	6 (26.1%)	0.279	15.3 (11.1, 17.3)	0.217
Poor outcome*	Yes	84 (43.5%)	**0.016**	20 (23.8%)	0.159	14.6 (12.1, 18.3)	**<0.001**
Contralateral side							
Recanalization	Yes	77 (23.9%)	0.393	13 (16.9%)	0.217	10.3 (7.2, 13.2)	0.348
sICH	Yes	16 (44.4%)	**0.003**	3 (18.8%)	0.615	10.1 (8.0, 11.7)	0.351
Poor outcome*	Yes	54 (28.0%)	**0.036**	13 (24.1%)	**0.030**	11.3 (9.3, 13.2)	**0.002**

NRS, napkin-ring sign; PR, Positive remodeling; sICH, symptomatic intracranial hemorrhage. * Data availability: Outcome at 3 months were unavailable for 9 patients (lost to follow-up). Bold values indicate statistical significance at *p* < 0.05.

**Table 3:** Association of napkin-ring sign plaques and poor outcome after adjusted to clinical parameters.

Characteristics	Symptomatic side		Contralateral side	
	OR (95% CI)	*P*	OR (95% CI)	*P*
NRS plaques	1.636 (1.001, 2.676)	0.050	1.713 (0.966, 3.039)	0.066
PR of NRS	2.557 (0.910, 7.181)	0.075	3.314 (0.709, 5.496)	0.128
NRS area	1.285 (1.150, 1.435)	**<0.001**	1.260 (1.033,1.536)	**0.022**

Each parameter was adjusted for age, sex, sit of occlusion, NIHSS at baseline, and intravenous thrombolysis. NRS, napkin-ring sign; PR, Positive remodeling; NIHSS, National Institutes of Health Stroke Scale. Bold values indicate statistical significance at *p* < 0.05.

Table 2 reports that 322 (91.7%) patients achieved successful recanalization (mTICI ≥ 2b), whereas 29 (8.3%) did not. On the symptomatic side (Table 2), patients without recanalization had a higher percentage of NRS plaques (18 [62.1%] vs. 114 [35.4%], *p* = 0.005) and a higher percentage of PR (7 [38.9%] vs. 19 [16.7%], *p* = 0.036) than those of patients with recanalization. On the contralateral side, no substantial difference was found based on recanalization status. After receiving EVT, 36 (10.3%) patients experienced sICH, and we found a high percentage of NRS plaques on both the symptomatic side (23 [63.9%] vs. 109 [34.6%], *p* = 0.001; Table 2) and the contralateral side (16 [44.4%] vs. 70 [22.2%], *p* = 0.003).

As shown in Table 4, the percentage of NRS plaques on the symptomatic side was higher than that on the contralateral side in all patients (132 [37.6%] vs. 86 [24.5%], *p* < 0.001). In the subgroup analyses, the percentage of NRS differed in patients with LAA stroke (74 [48.7%] vs. 42 [27.6%], *p* < 0.001) but not in patients with CE (49 [31.2%] vs. 44 [28.0%], *p =* 0.537). In addition, the NRS area was higher on the symptomatic side than on the contralateral side (13.1 [9.0, 17.3] vs. 10.6 [7.5, 13.2], *p* = 0.001 in all patients; 12.7 [8.7, 18.2] vs. 10.6 [6.7, 13.2], *p* = 0.018 in patients with LAA stroke; and 14.1 [9.6, 16.7] vs. 10.4 [8.1, 13.1], *p* = 0.011 in patients with CE stroke). For PR, no difference was observed between the symptomatic and contralateral sides.

**Table 4:** Comparison of the plaque with napkin-ring sign counts between symptomatic and contralateral sides.

Characteristics	All patients			LAA stroke patients			CE stroke patients		
	Symptomatic	Contralateral	*P*	Symptomatic	Contralateral	*P*	Symptomatic	Contralateral	*P*
NRS plaques *N* (%)	132 (37.6%)	86 (24.5%)	**<0.001**	74 (48.7%)	42 (27.6%)	**<0.001**	49 (31.2%)	44 (28.0%)	0.537
PR of NRS *N* (%)	26 (19.7%)	16 (18.6%)	0.862	18 (24.3%)	11 (26.2%)	0.823	7 (14.3%)	5 (11.4%)	0.675
NRS area mm^2^	13.1 (9.0, 17.3)	10.6 (7.5, 13.2)	**0.001**	12.7 (8.7, 18.2)	10.6 (6.7, 13.2)	**0.018**	14.1 (9.6, 16.7)	10.4 (8.1, 13.1)	**0.011**

LAA, Large-artery atherosclerosis; CE, Cardioembolism. NRS, napkin-ring sign; PR, Positive remodeling. Bold values indicate statistical significance at *p* < 0.05.

## Discussion

Our study revealed that NRS plaques were more frequent in patients with LAA than in those with CE, and they often appeared on the symptomatic side. We found that patients without recanalization had a high percentage of NRS plaques on the symptomatic side, and patients with sICH had a high percentage of NRS plaques on both sides. A larger plaque area was associated with worse outcomes. Therefore, observing plaque morphology on CTA before EVT may help us better predict outcomes.

When we calculated NRS plaque area in all patients, we found greater areas on both the symptomatic and contralateral sides in patients with poor outcomes. Even after adjusting for other clinical parameters, the difference remained statistically significant. NRS plaques identified on CCTA refer to advanced lesions, characterized by a large necrotic core and a large fibrous component associated with neovascularization (26), both of which are prone to rupture (27). This finding aligns with our observation that larger plaque areas were associated with worse outcomes. Thus, it can be concluded that the presence of NRS plaques may be associated with poor prognosis, and the greater the plaque area, the worse the prognosis. However, a single examination cannot capture the entire process of arteriosclerosis, and further research is needed to confirm this hypothesis.

We found that patients who experienced sICH after EVT had a high percentage of symptomatic and contralateral NRS plaques. sICH has been reported to be associated with hyperglycemia, and diabetes mellitus is more likely to be associated with a worse 3-month outcome in patients who underwent EVT (28, 29). Individuals with diabetes are more likely to develop lipid-rich atherosclerosis, known as vulnerable plaques, which can lead to arterial thrombosis (30). In addition, other studies suggest that atherosclerosis and poor collateral circulation are associated with sICH and worse outcomes (31, 32). These findings suggest that poorer vascular conditions may increase the likelihood of sICH, and NRS plaques indicate poor vascular conditions. Therefore, the high proportion of sICH in patients with NRS plaques may be related to this, highlighting the need to consider postoperative sICH when encountering NRS plaques before EVT and to prepare for possible risks in advance.

An NRS plaque is defined as one of the high-risk plaque features that is associated with cholesterol crystals and thin-cap fibroatheroma, is more prone to rupture, and may cause further inflammatory reactions (33, 34). In addition, NRS plaques on CT have significant histopathological features of coronary atherosclerotic plaques, characterized by a large necrotic core and a large fibrous component often associated with neovascularization (5). Because these features are associated histologically with advanced atherosclerosis and rupture-prone lesions, the NRS may be a marker for advanced disease on coronary CT angiography (CCTA). NRS plaques detected on CCTA have a high specificity and positive predictive value for the presence of advanced lesions (35), and intensification of preventive anti-atherosclerosis therapy seems to be warranted.

Although many studies have reported a number of independent prognostic predictors for patients who underwent EVT (36–40), there are no reports on whether NRS plaques are associated with the prognosis of patients after EVT. One study has shown that an increased incidence of NRS on CTA is positively correlated with the occurrence of AIS, which led us to investigate the presence of NRS plaques in patients with AIS. In our study, the association between baseline NIHSS score and functional outcome is consistent with established literature (16). A key objective was to evaluate whether imaging markers, particularly the napkin-ring sign, retain predictive significance after accounting for NIHSS and other clinical predictors. In the multivariable logistic regression analysis (Table 4), baseline NIHSS score was included as a covariate. The results demonstrated that the napkin-ring sign remained an independent determinant of functional outcome in models adjusting for baseline NIHSS, indicating that it contributes prognostic information beyond initial stroke severity. In the mean time, our study found that patients with LAA had a high percentage of NRS plaques, indicating that they had worse vascular conditions. Additionally, the percentage of NRS plaques on the symptomatic side was higher than that on the contralateral side, especially in patients with LAA, which strengthens our confidence in using the vascular conditions on the symptomatic side as indicators of disease severity.

We discovered that the percentage of NRS plaques and the percentage of PR on the symptomatic side were associated with recanalization in EVT, whereas no difference was observed on the contralateral side. NRS plaques often represent a greater burden than non-NRS plaques, and they are advanced lesions associated with neovascularization and inflammatory processes. Daisuke Kinoshita et al. concluded that four features, including NRS and PR, were associated with all optical coherence tomography features of plaque vulnerability (7); both features indicate poor vessel conditions. During the procedure, the presence of NRS plaques in the vascular access may also affect the outcome of the operation, mainly because they are vulnerable plaques and can be easily disrupted during EVT. This finding suggests that we need to take extra care when selecting the vascular access route. Therefore, we speculated that the percentage of NRS plaques could serve as an indicator of risk after revascularization.

Our study has several limitations. First, this was a retrospective, single-center study; hence, biases may be present in the study process. Moreover, owing to the relatively small sample size, establishing causation may not be fully possible. Further multi-centre collaborations and randomized trials with larger datasets should be conducted to confirm our results. Second, a key limitation of this study is that, due to the inherent constraints of CTA, we were unable to formally assess the incremental prognostic value of NRS beyond established plaque scoring systems. This is because CTA cannot evaluate key imaging features, such as intraplaque hemorrhage, which are required to construct a standard Plaque-RADS classification. Furthermore, our patient cohort was confined to those with anterior circulation lesions. The applicability of NRS to posterior circulation lesions therefore remains to be explored in future studies. Third, assessing the NRS throughout the entire vessel, not limited to the common carotid artery, may be more relevant, and future prospective studies examining the whole vessel may help us assess prognosis more comprehensively.

In conclusion, our study suggests that the appearance of NRS may be associated with reduced recanalization, increased sICH, and poor outcomes, and the NRS area may be an independent risk factor for poor outcomes in patients with AIS. We believe that this study has the potential to lay the groundwork for larger evaluations to further validate our findings and address this important problem. Preoperative observation of vascular plaque morphology, detection of NRS plaques, and estimation of plaque area may enable us to predict outcomes in patients with AIS undergoing EVT.

## Data Availability

The original contributions presented in the study are included in the article/Supplementary material, further inquiries can be directed to the corresponding authors.

## Supplementary material

SUPPLEMENTARY FIGURE S1 This figure includes two other patients with relatively mild symptoms. The top two images are from an 80-year-old woman diagnosed with scattered infarcts in the right cerebral hemisphere. The bottom two images are from a 78-year-old man diagnosed with an infarct in the left basal ganglia. The yellow round demonstrates the low attenuation area of the plaque surrounded by a high-attenuation rim (blue round), and the red area represents the lumen field with contrast.
