# Supplementary figures and images for: Napkin-ring sign plaques are associated with clinical outcome in patients with acute ischemic stroke after endovascular therapy
Source: Front Neurol. 2026 Jun 11;17:1792239. doi: 10.3389/fneur.2026.1792239 (PMC13293798; doi:10.3389/fneur.2026.1792239)

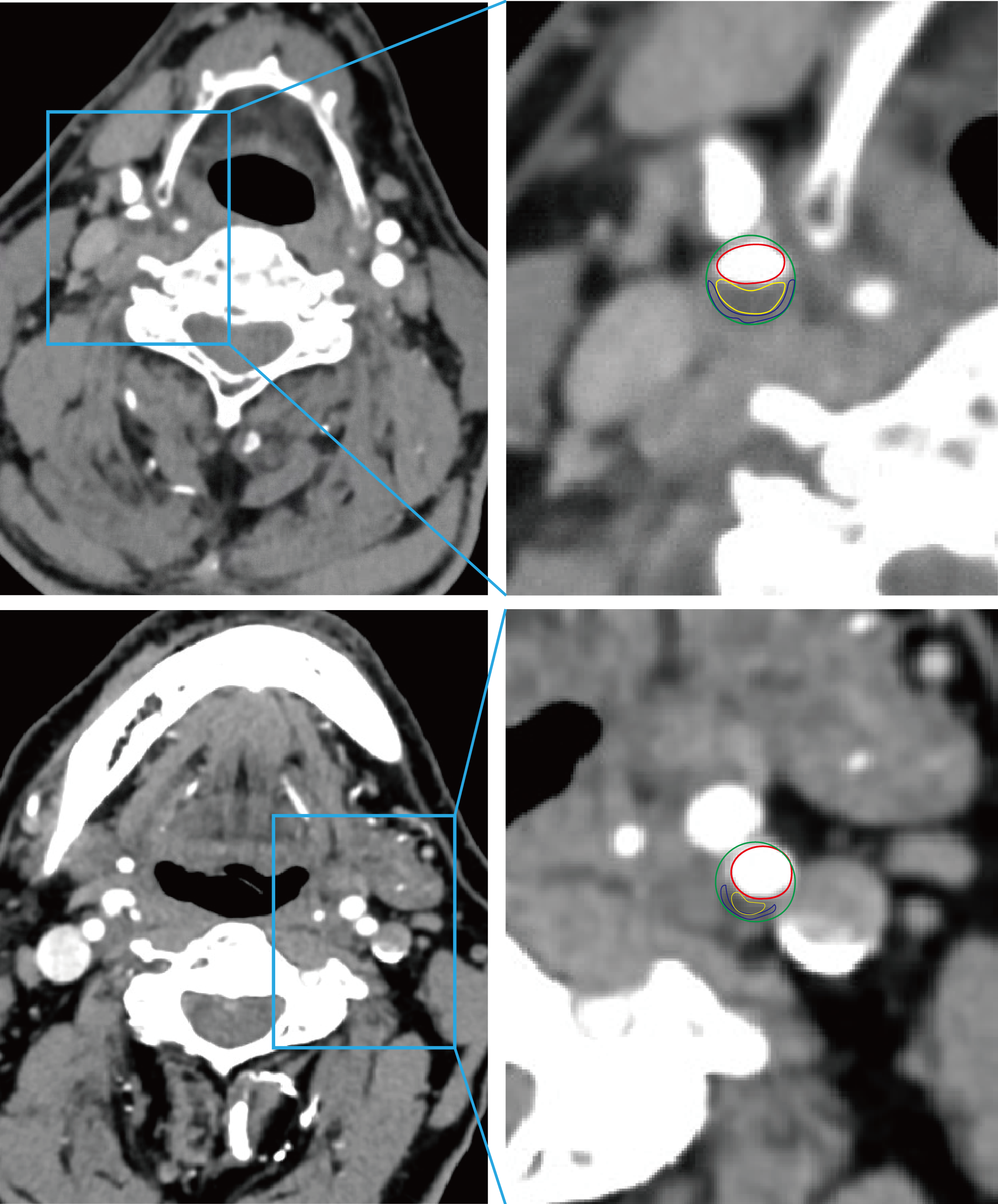

Supplement: SUPPLEMENTARY FIGURE S1 — This figure includes two other patients with relatively mild symptoms. The top two images are from an 80-year-old woman diagnosed with scattered infarcts in the right cerebral hemisphere. The bottom two images are from a 78-year-old man diagnosed with an infarct in the left basal ganglia. The yellow round demonstrates the low attenuation area of the plaque surrounded by a high-attenuation rim (blue round), and the red area represents the lumen field with contrast. [file Image_1.TIF]
